# Draft Genome Sequence of an Aflatoxin-Producing Aspergillus flavus Strain Isolated from Food

**DOI:** 10.1128/mra.00894-21

**Published:** 2022-02-03

**Authors:** Alexandra Schamann, Rolf Geisen, Markus Schmidt-Heydt

**Affiliations:** a Max Rubner-Institut, Federal Research Institute of Nutrition and Food, Department of Safety and Quality of Fruit and Vegetables, Karlsruhe, Germany; Vanderbilt University

## Abstract

Aspergillus flavus is the main producer of carcinogenic aflatoxins and thus is one of the most important fungal food contaminants. Here, we report that the genome of A. flavus strain MRI19 was sequenced using MiSeq and PacBio platforms and that a hybrid assembly was generated.

## ANNOUNCEMENT

The filamentous fungus Aspergillus flavus contaminates economically important crops like maize and peanuts as a preharvest and postharvest contaminant, causing large economic losses (1, 2). Additionally, due to its production of hepatotoxic aflatoxins and neurotoxic cyclopiazonic acid, A. flavus is a threat to food safety in regions with warm and humid climates that are affected by high levels of contamination, such as Sub-Saharan Africa (3). To analyze gene clusters encoding secondary metabolites, the whole genome of A. flavus strain MRI19 was sequenced using MiSeq and PacBio platforms.

**FIG 1:**
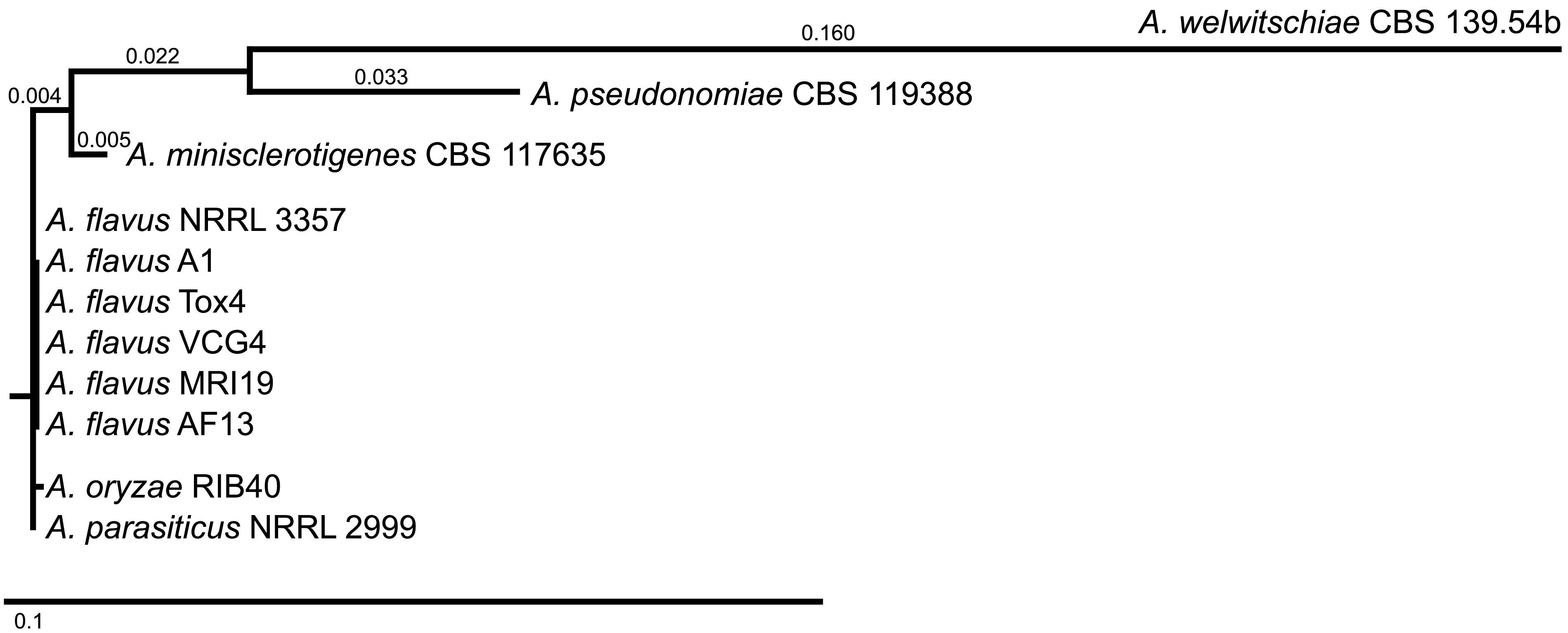
Phylogenetic analysis of Aspergillus strains. Identification of strain A. flavus MRI19 was based on sequencing of PCR products of the ITS region (ITS1/ITS4 primers) and the β-tubulin (Bt2a/Bt2b) and calmodulin (cmd5/cmd6) genes (4–6). PCR was performed using the peqGOLD *Taq* DNA polymerase all-inclusive kit (VWR International GmbH, Darmstadt, Germany) with the recommended mastermix composition, 2.5 μL of each primer (5 pmol/μL), and 5 μL DNA. The following cycling program was used: 95°C for 3 min; 40 cycles of 95°C for 30 s, 60°C (β-tubulin and calmodulin) or 50°C (ITS) for 40 s, and 72°C for 90 s; and 72°C for 3 min. Forward and reverse sequences were assembled using SeqMan Pro (LaserGene v16). Consensus sequences were compared at GenBank using BLASTN. For generation of the phylogenetic tree, the three sequences were concatenated and were compared to those of a variety of other Aspergillus strains as examples (A. flavus AF13, GenBank accession no. CP059863.1, CP059859.1, and CP059864.1 [13]; A. flavus A1, GenBank accession no. CP051064.1, CP051060.1, and CP051065.1 [14]; A. flavus NRRL 3357, GenBank accession no. CP044623.1, CP044622.1, and CP044617.1 [15]; A. flavus Tox4, GenBank accession no. CP051048.1, CP051044.1, and CP051049.1 [14]; A. flavus VCG4, GenBank accession no. CP051056.1, CP051052.1, and CP051057.1 [14]; Aspergillus oryzae RIB40, GenBank accession no. NC_036440.1, NC_036436.1, and NC_036441.1 [16]; Aspergillus parasiticus NRRL 2999, GenBank accession no. CP051032.1, CP051028.1, and CP051033.1 [14]; Aspergillus minisclerotigenes CBS 117635, GenBank accession no. ML732812.1, ML732765.1, and OL711675.1 [17]; Aspergillus pseudonomiae CBS 119388, GenBank accession no. NW_022476058.1, NW_022476011.1, and OL711683.1 [17]; Aspergillus welwitschiae CBS 139.54b, GenBank accession no. NW_020798696.1, NW_020798709.1, and OL711714.1 [18]), with the same fragments of the β-tubulin and calmodulin genes and the ITS region being concatenated. All sequences were aligned using MUSCLE (MegAlign Pro). A phylogenetic tree was generated using the neighbor-joining algorithm.

The strain was isolated from tiger nuts grown in the surroundings of Valencia, Spain, via dilution series and cultivation on selective nutrient media. Strain identification was based on microscopic morphological determination and partial Sanger sequencing by Eurofins Genomics (Cologne, Germany) of PCR products of the internal transcribed spacer (ITS) region and the β-tubulin and calmodulin genes (Fig. 1) (4–6). For isolation of genomic DNA (gDNA) for MiSeq sequencing, A. flavus was grown for 7 days at 25°C in YES broth (20 g/L yeast extract, 150 g/L saccharose); gDNA was extracted from the pure culture using the NucleoSpin plant II kit (Macherey-Nagel, Düren, Germany). For extraction of gDNA for PacBio sequencing, the strain was grown on potato dextrose agar (PDA) at 25°C for 4 days. The mycelium was homogenized in liquid nitrogen using a mortar and pestle, and lysis was achieved with cetyltrimethylammonium bromide (CTAB) buffer (0.1 M Tris [pH 8.0], 1.4 M NaCl, 20 mM EDTA, 2% [wt/vol] CTAB, 4% [wt/vol] polyvinylpyrrolidone with an average molecular weight of 10,000 [PVP-10]). Phenol-chloroform was used for extraction, and propan-2-ol and 7.5 M ammonium acetate were added overnight for precipitation. Different DNA extraction methods were applied for the two sequencing platforms due to different requirements regarding DNA quality and quantity, which were verified using a NanoDrop 1000 spectrophotometer and a Qubit 3.0 fluorometer (both from Thermo Fisher Scientific GmbH, Bremen, Germany), respectively.

Paired-end short-read genome sequencing was carried out on a MiSeq instrument (Illumina, San Diego, CA, USA) using a sequencing library generated with the Nextera DNA Flex library preparation kit (Illumina) and quality checked with the Experion DNA 1K analysis kit (Bio-Rad, Feldkirchen, Germany). Based on the quality control (FastQC v0.11.3) of the 300-bp-long raw reads (18,887,287 read pairs), reads were quality trimmed (Trimmomatic v0.39) (7). Long-read PacBio genome sequencing was performed on a Sequel platform by BGI (Hong Kong, China), resulting in 114,583 total reads (*N*_50_, 9,775 bp). Quality was checked using LongQC v1.2.0 (8).

Default software parameters were used except where otherwise noted. *De novo* hybrid assembly was carried out with SPAdes v3.14.1, resulting in 68 contigs after the elimination of short contigs (<400 bp) and mitochondrial sequences (9, 10). The assembly size was 36.78 Mb, with a coverage of 75-fold. The average GC content was 48.36%, the *N*_50_ value was 1.46 Mb, and the *L*_50_ value was 10. Secondary metabolite biosynthetic gene clusters (BGCs) were predicted with antiSMASH v6.0.1, choosing the cluster finder algorithm for BGC border prediction (11, 12). In total, 46 BGCs were identified. In further analysis, the genome of A. flavus will be compared to that of other aflatoxin-producing Aspergillus species to find species-specific characteristics.

### Data availability.

This whole-genome shotgun project has been deposited in DDBJ/ENA/GenBank under accession no. JAGYXF000000000 and BioProject accession no. PRJNA727175. The version described in this paper is version no. JAGYXF010000000. The raw sequence reads have been deposited in the Sequence Read Archive (SRA) under accession no. SRX10945236 and SRX10945235. The partial gene sequences have been deposited in GenBank under accession no. OL513390, OL513391, and OL513392.
